# Safety Assessment of *Ocimum Basilicum* Hydroalcoholic Extract in Wistar Rats: Acute and Subchronic Toxicity Studies

**Published:** 2012

**Authors:** Hamid Reza Rasekh, Leila Hosseinzadeh, Soghra Mehri, Mohammad Kamli-Nejad, Majid Aslani, Farahnaz Tanbakoosazan

**Affiliations:** 1 *School of Pharmacy, Shahid Beheshti University of Medical Sciences, Tehran, Iran*; 2 *School of Pharmacy, Kermanshah University of Medical Sciences, Kermanshah, Iran*; 3 *School of Pharmacy, Mashhad University of Medical Sciences, Mashhad, Iran*

**Keywords:** Acute toxicity, Ocimum basilicum, Rats, Subchronic toxicity

## Abstract

**Objective(s):**

*Ocimum basilicum* L*. *is widely used in folk medicine of many countries including . Both *O.* *basilicum* and its oil extract have received considerable attention for their potential medicinal properties, but there are a few reports about possible toxicity of this plant. Therefore, in the present study, acute and subchronic toxicity of *O. basilicum* hydroalcohlic extract have been evaluated in Wistar rats.

**Materials and Methods:**

For the acute toxicity assessment, five groups of 10 animals (5 male, 5 female) received four different single dose of extract orally, the animals were, then, kept under observation for 14 days. For subchronic toxicity, the animals were divided into four groups (5 male, 5 female) and were gavaged daily by 50, 200 and 500 mg/kg of extract. Mortality, clinical signs, body weight changes, food and water consumption, and hematological and biochemical parameters were monitored during the study period. On the 45th day, animals were sacrificed and gross findings, weight of liver and left kidney and liver histological markers were assessed.

**Results:**

The results of acute study indicated that LD_50 _of *O. basilicum* is higher than 5 mg/kg. In subchronic study, no adverse effects were observed on serum parameters in male and female rats. The hematological results showed a reduction in the hematocrit, platelets and RBC in both sexes. No abnormalities were observed in other parameters.

**Conclusion:**

Based on the results of this study, present data suggest that hematologic system could serve as a target organ in oral toxicity of this plant.

## Introduction

*Ocimum basilicum* L. is a plant belonging to the Lamiaceae family. This plant is cultivated in large quantities in different regions of and is widely used in traditional Iranian medicine as a culinary herb and as a well known source of flavoring principles (1). Furthermore both *O.* *basilicum* and its oil extract have received considerable attention for their potential therapeutic properties. These include hypoglycemic, hypolipidimic (2), antiulcerogenic (3), antimicrobial (4), chemopreventive (5), anti-mutagenic (6), anti-oxidant (7) and antihypertensive (8) effects. It has also been used as a folk remedy to treat various ailments such as; feverish illnesses, poor digestion, nausea, abdominal cramps, gastro-enteritis, migraine, insomnia, depression, gonorrhoea, dysentery, and chronic diarrhoea exhaustion (9). 

There are a few reports about possible toxicological actions of *O. basilicum* (10). In this study, acute and subchronic oral dose toxicity of *O.* *basilicum* hydroalcholic extract were evaluated to investigate the potential toxicity in Wistar rats using hematological and histopathological examinations, as well as, biochemical parameters.

## Materials and Methods

***Plant materials***

The aerial parts of the plant was collected from Shahr-e-Ray in the south and dried in shed. The plant was authenticated by Department of Pharmacognosy and a voucher specimen (Registration Number 1259) and a sample of the plant has been deposited in the Herbarium of the of ( , ). The plant sample was first ground to a fine powder and soaked in ethanol-water (80-20) at room temperature. After 96 hr, the sample was centrifuged and the supernatant was collected. The solvent was evaporated with RapidVap vaccum evaporation system at 40 °C. Dried extract were stored at 4 °C until using. In this way, 100 g of dried plant yielded 20 g of extract, which was used for the experiment. 

***Evaluating toxicity following single dose administration***

***Animals***

Four weeks old Wistar rats of both sexes were purchased from Razi Research Institute (Hesarak, ) and acclimated to holding facilities for 2 weeks prior to dosing. Animals were randomly assigned to control and four treatment groups (5 rats/sex group) and were housed in clear plastic cages containing wood shavings for bedding. Each cage contained five rats of the same sex and were fed on normal laboratory chow (Pars Co., ) and given tap water *ad libitum *throughout the study. Environmental conditions were maintained at a temperature of 23±2 ^◦^C and a relative humidity of 40±10% with 12 hr light/dark cycle. At the onset of dosing, males weighed 164±19 g and females weighed 142±15 g. The research was conducted in accordance with the internationally accepted principles for laboratory animals’ use and care as found in the US Guidelines (NIH Publication no. 85–23, revised in 1985). 

***Administration***

Animal were fasted 4 hours prior to dosing. The extract was dissolved in saline. Treatment groups were administrated by oral gavage at doses of 50, 500, 1000 and 2000 mg/kg body weight. The control group received saline by gavage in the same volume. All solutions were freshly prepared just prior to dosing and were tightly capped.

***Observations***

Mortality and clinical signs were monitored continuously for 10 hr after dosing for signs of toxicity after dosing on day one. For the rest of the 14 days of the period of study, animals were monitored daily for mortality and any changes in food and water consumption, and any additional behavioural or clinical signs of toxicity. Animals’ body weights were measured prior to dosing and on days 7 and 14. On day 14, all animals were killed and at the end of the study the number of dead animals were expressed in percentage and, if possible, the LD_50_ value was established using Probits method (11).

***Evaluation of toxicity following subchronic treatment***

***Group assignment and treatment***

Animals were caged randomly in clear plastic cages. When dosing was initiated, males weighed 200±19 g and females weighed 161±10 g. Animals were divided into four dose groups (5 rat/ sex group). The first group was given 1 ml tap water and taken as a control. The second, third and forth groups were given a single dose of 50, 200, 500 mg/kg of *O.* *basilicum* by gavage daily. All solutions were freshly prepared just prior to dosing and were tightly capped. 

***In life observations***

Observations of mortality and toxicological signs were made daily for 45 days. The time of onset, intensity and duration of these symptoms, if any, were recorded. The weight of each rat was recorded on day 0 and at weekly intervals throughout the course of the study. Food and water consumption were measured three times a week.

***Biochemical and hematology analysis***

After 45 days, while fasted for about 12 hr, the animals were anaesthetized with an i.p. injection of a mixture of ketamine (40 mg/kg) and xylazine (10 mg/kg). The jugular vein was exposed, and blood samples were taken by jugular vein puncture (12). The following hematological parameters were determined by Sysmex K-1000 fully automated hematology analyzer: Erythrocyte (RBC), total and differential leukocyte (WBC), hematocrit (Hct), hemoglobin (Hb), platelet count, mean corpuscular volume (MCV), mean corpuscular hemoglobin (MCH), mean corpuscular hemoglobin concentration (MCHC), mean platelet volume (MPV), platelet distribution width (PDW), and red distribution width (RDW). Blood samples for biochemical analysis were centrifuged at 3000×g for 5 min and the serum was collected and analyzed for glucose, uric acid, creatinine, albumin, cholesterol, low density lipoprotein (LDL), high density lipoprotein(HDL), very low density lipoprotein (VLDL), triglycerides, aspartate aminotransferase (SGOT), alanine aminotransferase (SGPT) blood urea nitrogen(BUN), total protein and lactate dehydrogenase (LDH) . These levels were determined by COBAS Mira S chemistry analyzer (Roche Diagnostic Systems).

***Necropsy***

Following blood collection, rats were sacrificed by decapitation. The macroscopic appearance of organs was noted and weight of liver and left kidney was recorded. Livers were preserved in 10% neutral buffered formalin for microscopic histopathological examinations. The tissue was embedded in paraffin, sectioned, stained with haematoxylin-eosin and was examined microscopically.

***Statistical analysis***

Mean±SEM were calculated for body weights, food consumption, organ/body weight ratios, hematological and biochemistry factors. Differences between dose groups with controls were evaluated for males and females separately by performing one-way analysis of variance (ANOVA) followed by Tucky´s post test. *P* values of 0.05 or less were taken as significant. Since treatment-related animal deaths were not observed, LD_50_ values were not measured. 

## Results

***Acute study***

All rats treated with different concentrations of total extract of *O.* *basilicum* were alive during the 14 days of observations. Normal body weight gains were observed in males and females of all dose groups. No significant differences were observed between the vehicle control and treatment groups. No abnormal gross findings were observed in any animals. The oral acute toxicity of *O.* *basilicum* hydroalcoholic extract was, therefore, considered as unclassified, since a dose of 2 g/kg did not induce deaths or toxic symptoms. 

***Subchronic study***

***Body weight, food and water consumption and mortality***

**Figure 1:**
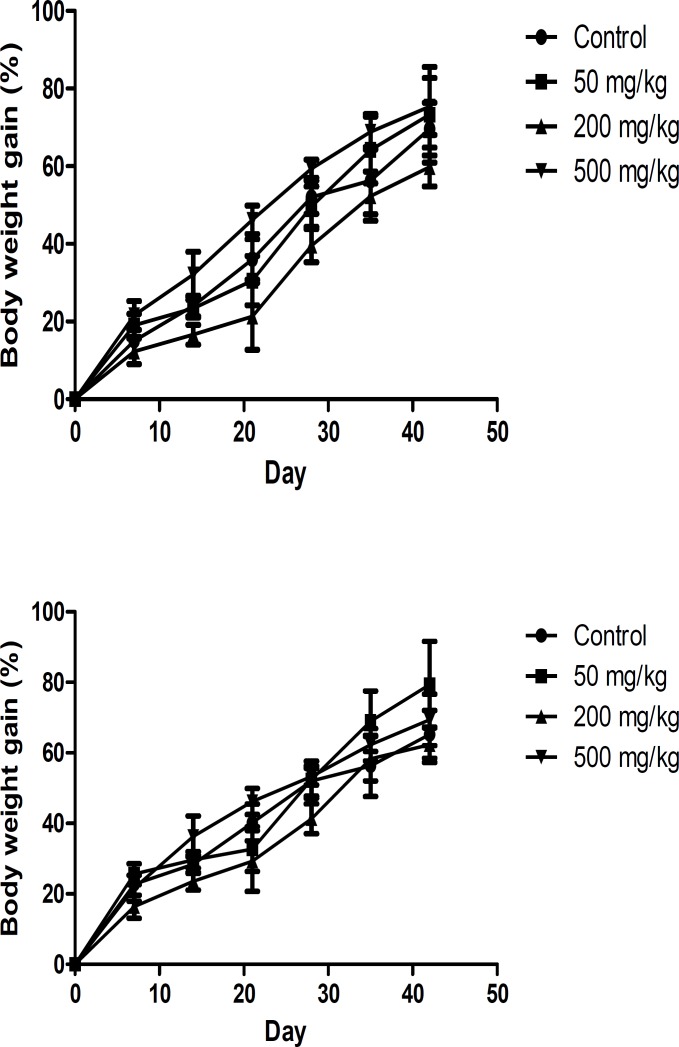
Changes in male (a) and female (b) rats body weight with duration of treatment. Each point represents mean±SEM, n= 5

**Figure 2:**
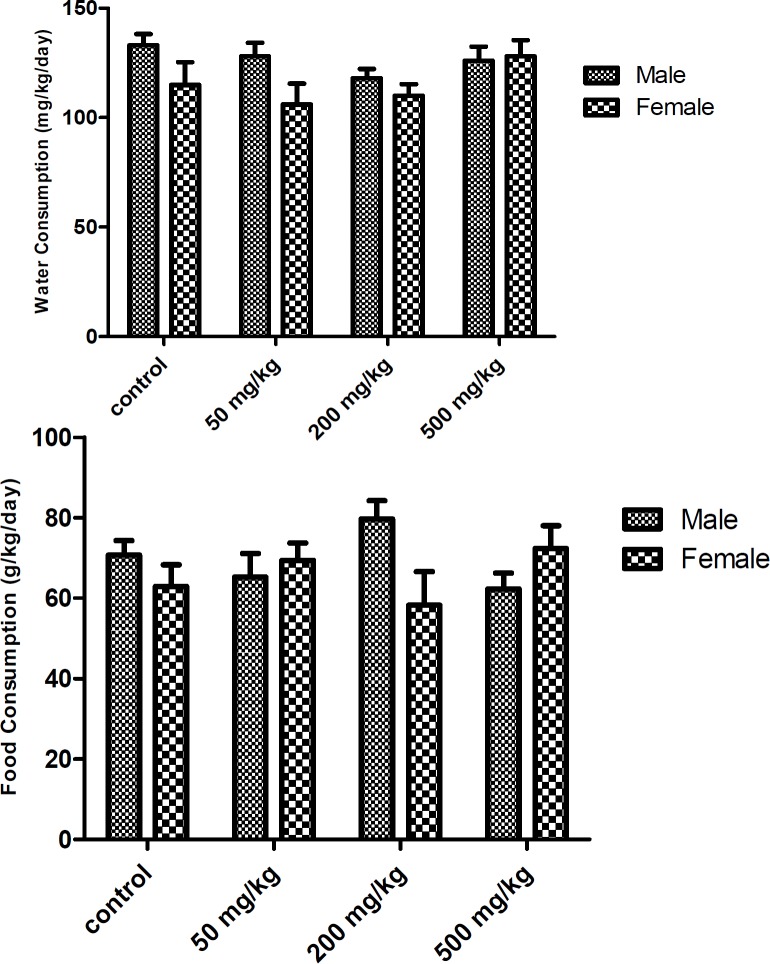
Food (a) and water (b) consumption of rats given *Ocimum basilicum* orally. Data represented as Mean±SEM, n= 5

There was no significant difference in body weights between control and treatment groups (Figure 1). There was no significant difference between food consumption of *O.* *basilicum* treated animals compared with control (Figure 2). No death was reported in any groups throughout the experimental period. 

***Hematological analysis***

Results of the hematological study are given in Table 1. The data showed that RBC count was decreased significantly in both sexes that received 500 mg/kg of *O.* *basilicum*. Platelets decreased significantly in all female rat groups and in the highest dose group of male rats. Moreover, Hb was reduced in all female treated groups and hematocrit was reduced significantly in the female groups that received 200 and 500 mg/kg and male rats that received 500 mg/kg of *O. basilicum* compared to control (Table 1). 

***Biochemichal analysis***

No adverse effect was observed on serum parameters in male and female rats on the 45th day. Serum cholesterol and creatinine were significantly decreased in female rats at 200 and 500 mg/kg Ocimum groups in comparison with control. Furthermore, HDL levels were elevated significantly among middle and high dose groups of female rats. Also, triglyceride, VLDL and LDL were significantly decreased in male high dose group. Other serum biochemical parameters remained unaffected following *O.* *basilicum* treatment (Table 2).

***Organ weight and liver histopathology***

Relative organ weight measurements data are presented in Table 3. There were no remarkable differences in relative weights of liver and left kidney.

For histopathological evaluation, inflammatory reaction, focal necrosis, hypertrophy of kupffer cells, fatty degeneration and sinusoidal congestion in the liver were assayed. The obtained results showed that hepatocellular necrosis and kupffer cells hypertrophy is lower in control group than in the low dose groups (50 mg/kg) of both sexes. Slight increase in hepatocellular necrosis was found at 500 mg/kg group of males, but these changes are non significant when compared with control. No significant changes were observed in other parameters as compared with that of the control group.

**Table 1:** Hematological parameters of Wistar rats after 45 days treatment to *Ocimum basilicum*

		WBC	RBC	Hb	HCT
Sex	Dose mg/kg	(10^5 ^/*mm*^3^)	(10^6 ^/*mm*^3^)	*(g%)*	(%)
	Control	4.3±0.9	7.99±0.1	15.1±0.24	41.2±0.46
Male	50	4.3±1.4	7.9±0.05	15.2±0.1	40.37±0.45
	200	4.1±0.9	7.75±0.1	14.6±0.09	39.5±0.42
	500	3.2±0.07	7.45^**^±0.1	13.87±0.6	38.57^*^±0.8
	Control	2.9±0.49	7.46±0.1	14.77±0.1	39.1±0.6
Female	50	2.8±0.25	7.1±0.07	14.36^**^±0.02	37.46±0.2
	200	2.1±0.45	7±0.2	13.6^**^±0.1	36.45^*^±0.4
	500	2.1±0.06	6.9^*^±0.1	13.3**±0.07	36.4^*^±0.4

Data given as mean ±SEM for =5. *P<0.05, **P<0.01, vs. control

**Table 2:** Biochemical parameters of Wistar rats after 45 days treatment with *Ocimum basilicum*

		Blood sugar	Creatinine	Uric Acid	Triglycerides
Sex	Dose mg/kg	mg%	mg%	mg%	mg%
	Control	161±9	0.5±0.05	2.5±0.35	188±13.5
Male	50	136.3±14	0.56±0.05	2.5±0.15	149.5±18.3
	200	149.5±11	0.6±0.05	1.97±0.05	139±18
	500	160.5±14.45	0.45±0.05	1.95±0.15	106.75^*^±22.1
	Control	155±3.6	0.73±0.025	1.7±0.5	122.3±15.5
Female	50	145.66±2.3	0.52±0.05	1.82±0.04	113.25±7.4
	200	145±18.5	0.52±0.025	1.92±0.04	104.25±12
	500	138.5±21	0.4^*^±0.13	1.79±0.17	96±13.4

**Table 3:** Biochemical parameters of Wistar rats after 45 days treatment with *Ocimum basilicum* (Continued)

	Dose	BUN	Total protein
Sex	mg/kg	mg%	mg%
	Control	22.6±1.5	6.66±0.15
Male	50	2.8±1	6.5±0.25
	200	19.17±3.4	6.5±3.6
	500	19.16±1.02	6.4±0.04
	Control	19.16±1.02	7.26±0.25
Female	50	17.6±1	7.2±0.15
	200	18.57±1.3	7.06±0.1
	500	17.1±0.9	6.67±0.09

Data given as mean±SEM for =5. *P< 0.05, **P< *0.01, *vs. control

**Table 4:** Organ weight of Wistar rats after 45 days treatment with *Ocimum Basilicum*

Animal groups	Male		Femaleale	
	Liver (%)	Kidney (%)	Liver (%)	Kidney (%)
Control	2.5 ± 0.22	0.31± 0.02	2.48 ± 0.07	0.33 ± 0.01
50 ) mg/kg )	2.9± 0.4	0.31 ± 0.01	2.71 ± 0.25	0.35 ± 0.01
200 ( mg/kg )	2.7 ± 0.07	0.36 ± 0.01	2.81 ± 0.28	0.36 ± 0.01
500 )mg/kg )	2.87 ± 0.07	0.34 ± 0.01	2.62 ± 0.18	0.36 ±0.025

Data presented as mean ±SEM. for n= 5

## Discussion

Toxicity tests are not designed to reveal the safety of a chemical, but to characterize the toxic effects a chemical can produce (13). They are essentially performed on either mice or rats because of their availability, low cost and the wealth of toxicology data in the literature which is already available for these species. The first toxicity test performed on a compound is the evaluation of acute toxicity, determined from the administration of a single exposure (13). 

Based on common classification of the relative toxicity of chemicals, acute toxicity results indicated that *O.* *basilicum* hydroalcoholic extract was classified as a non toxic substance because the oral LD_50_ was greater than 5000 mg/kg. Previously, acute and subacute toxicity of *O. basilicum* essential oils (5-1000 mg/kg) have been investigated. It was established that rats given 5–1000 mg/kg body weight of the essential oil, survived all with no abnormalities in their general behavior throughout the 14 days of study (11).

 The principle goals of subchronic study are to further identify and characterize the specific organ or organs that maybe affected by test compound after repeated administration (13).

 Generally, the reductions in body weight gain and internal organ weights are simple and sensitive indices of toxicity after exposure to toxic substances. The gavage of *O.* *basilicum* up to 500 mg/kg to male and female Wistar rats for 45 days was not associated with any mortalities and abnormalities in general conditions, behavior, growth, and food and water consumption of animals. Additionally, treatment with *O. basilicum* extract did not produce any statistically significant difference in both body and organ weights. 

Hematological studies easily reveal anomalies in body metabolic processes, and the blood profile usually furnishes vital information on the response of the body to injury, deprivation and/or stress (14). In the present study we observed a reduction in the hematocrit, palates and RBC in the both sexes and hemoglobin in the female rats. The reduction of hematocrite and hemoglobin levels indicates a possible effect on the integrity of red blood cells. Thrombocytopenia may be due to decreased production or increased destruction of platelets. The probable mechanism by the help of which the *O.* *basilicum* produces reduction in platelets count is the suppression of the bone marrow, a condition that may also lead to the suppression of committed granulocytic precursors (14). However, this is not an established hypothesis and more investigations need to be done. Interestingly, other studies have also shown that *O. gratissimum* and *O. suave*, two other species of* Ocimum*, are able to decrease platelets in rats (15, 16). 

In this study, minor changes were noted in some of biochemichal parameters in *O. basilicum* extract - treated groups. A statistically significant deacrease in LDL, VLDL triglyceride and BUN were noted in male rats. In the female rats a significant decrease in cholesterol and a significant increase in HDL were noted. These effects maybe due to hypolipidemic effect of *O. basilicum* through the inhibition of the key enzymes in cholesterol and triglycerides synthesis or increasing cholesterol excretion throughout bile acid formation (2) and are not considered of any toxicological significant. 

It is clear that the liver plays a significant role in various metabolic processes; therefore, in this experiment liver histopathological studies were done. The result of microscopic examination of liver in control and treatment animals had showed no toxic effects on this organ and supported safety of *O.* *basilicum* data was inferred from biochemical parameters. 

## Conclusion

In conclusion, the subchronic oral administration of *O.* *basilicum* to Wistar rats did not cause death or any abnormal dose dependent changes in biochemical and liver histopathological parameters. A number of significant hematological changes were associated with the subchronic oral administration of *O. basilicum* to Wistar rats. These observations support the conclusion that hematologic system is a possible target organ for both sexes although the exact mechanisms are unclear. Based on the results of this study, it can be concluded that the risk of oral toxicity of *O.* *basilicum* on mammals is not negligible.
